# Mitigating *Salmonella* in Poultry Using Probiotics: Mechanisms, Challenges, and Opportunities

**DOI:** 10.3390/microorganisms14020365

**Published:** 2026-02-04

**Authors:** Oluwaseun D. Adeyemi, Samuel N. Nahashon

**Affiliations:** Department of Food and Animal Sciences, College of Agriculture, Tennessee State University, 3500 John A. Merritt Boulevard, Nashville, TN 37209, USA; oadeyemi@tnstate.edu

**Keywords:** *Salmonella*, probiotics, poultry, antimicrobial resistance

## Abstract

The global poultry industry continues to face significant challenges due to *Salmonella* infections, which pose severe public health concerns and economic losses. Recently, the reemergence of antimicrobial resistance has led to the restriction of antibiotic use in poultry, especially as growth promoters, thus accelerating the search for sustainable alternatives. Among these, probiotics have gained attention as potential candidates for improving poultry health and mitigating *Salmonella* colonization in the gut. This review summarizes the key mechanisms through which probiotics exert anti-*Salmonella* effects, including competitive exclusion, production of antimicrobial substances, reinforcement of the intestinal barrier, and modulation of host immune responses. Commonly used probiotic strains in poultry such as *Lactobacillus* and *Bacillus* are discussed, alongside emerging candidates derived from non-poultry hosts that may offer additional functional benefits. Despite encouraging findings, the use of probiotics in poultry faces several challenges, including strain-specific efficacy, variation in results across studies, environmental influences, and regulatory limitations. Therefore, we further explore future directions that are aimed at improving probiotic application in poultry production, such as microbiome-guided strain selection, advanced delivery systems, and combination therapies. Advancing our understanding of probiotic-pathogen-host interactions will be essential for optimizing probiotic use to enhance poultry health, reduce zoonotic transmission of *Salmonella*, and contribute to safer and more sustainable food systems.

## 1. Introduction

The global demand for poultry products continues to rise, driven by population growth, urbanization, and increased per capita meat consumption [1,2]. The poultry industry plays a pivotal role in global food security by providing animal protein including poultry meat and eggs which are among the most affordable sources of high-quality animal protein, especially in low- and middle-income countries. However, the productivity of the poultry industry is persistently challenged by *Salmonella* spp., a group of zoonotic, foodborne pathogens responsible for considerable public health concerns and economic losses [3]. According to the Centers for Disease Control and Prevention [4] over 1 million illnesses are estimated to be caused by non-typhoidal *Salmonella* annually in the United States alone, with contaminated poultry products often implicated as a primary source. In poultry, *Salmonella* mainly colonizes the intestinal tract, often without clinical symptoms, making detection and control difficult [5]. Birds act as asymptomatic carriers and shed the pathogen through feces which consequently contaminate the environment, feed, and eggs. The persistence of *Salmonella* in poultry production environments presents a significant challenge for on-farm biosecurity, processing hygiene, and food safety management. Historically, antibiotic growth promoters (AGPs) were widely used to control pathogens like *Salmonella* and enhance poultry growth performance. However, the global rise of antimicrobial resistance (AMR) and increasing consumer demand for antibiotic-free poultry products have prompted regulatory agencies in many countries to restrict or ban AGPs [6,7]. This shift has created an urgent need for sustainable alternatives to antibiotics that can support poultry health and reduce pathogen load without contributing to AMR.

Probiotics have emerged as one of the most promising strategies in this context. They are defined by the FAO/WHO [8] as “live microorganisms which, when administered and consumed in food in adequate amounts, confer a health benefit on the host”. Probiotics are gaining grounds and are increasingly being incorporated into poultry production to promote gut health, enhance immunity, and mitigate pathogen colonization, including *Salmonella* spp. Several recent studies have demonstrated the ability of specific probiotic strains to reduce *Salmonella* shedding and colonization in poultry through various mechanisms, such as competitive exclusion, modulation of the gut microbiota, enhancement of mucosal immunity, and production of antimicrobial metabolites [9,10]. While the use of probiotics in poultry production is not new, advancements in microbiome research, molecular tools, and high-throughput screening have enabled the development of more targeted and effective probiotic formulations. These innovations offer potential for tailoring probiotic interventions to specific poultry breeds, rearing systems, and pathogen profiles.

This review provides an update on the status of probiotic use against *Salmonella* in poultry with exploration into the types of probiotics used, their mechanisms of action, key findings from recent studies, and challenges associated with their commercial application. We also consider emerging probiotic candidates from other hosts or environments and highlight future directions for optimizing probiotic efficacy in poultry production systems.

## 2. Overview of Salmonella Prevalence and Pathogenesis in Poultry

**Figure 1:**
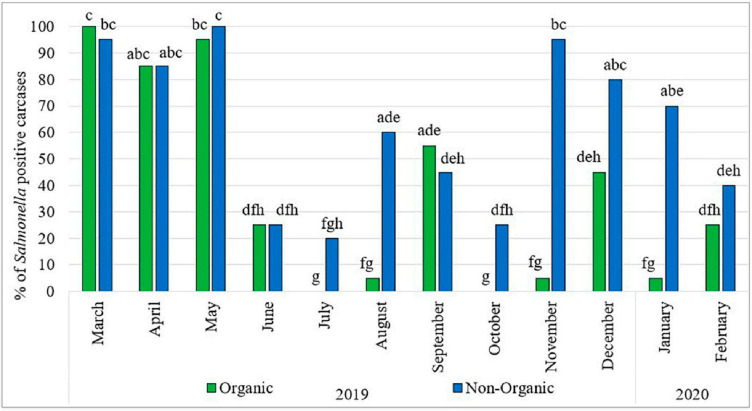
Prevalence of *Salmonella* in organic and non-organic chicken carcasses (2019–2020). Different lowercase letters (a–h) indicate significant differences in percentage values (*p* < 0.05) among months and chicken types. Adopted from Punchihewage-Don et al. [16] under CC BY license.

*Salmonella* spp. are Gram-negative, facultative anaerobic, rod-shaped bacteria within the family *Enterobacteriaceae* and among over 2600 known serovars, only a subset is commonly linked with poultry and human illness. The most prevalent serovars associated with poultry are *Salmonella enterica* serovar Enteritidis and *S. Typhimurium*, both of which significantly contribute to human salmonellosis globally [11]. Poultry can acquire *Salmonella* through multiple routes, including vertical transmission, from infected breeder hens to eggs and horizontal transmission via contaminated feed, water, litter, personnel, or equipment. In particular, chicks are highly vulnerable to early colonization, and once infected, they often remain asymptomatic carriers, shedding the pathogen intermittently throughout their lives [12]. Following oral ingestion, *Salmonella* colonizes the gastrointestinal tract, especially the cecum and ileum where it exhibits remarkable adaptability which allows it to adhere to and invade intestinal epithelial cells, persist in host tissues, evade immune responses, and form biofilms that promote environmental survival [13]. These adaptation strategies also allow *Salmonella* to persist in litter, dust, and farm infrastructure, making eradication a major challenge without robust biosecurity practices [14]. A study conducted in North Carolina, US found that 52.31% of broiler in commercial farms were infected with *Salmonella* serotypes [15] while multistate *Salmonella* outbreaks relating to backyard poultry have been recently discovered by the CDC [4]. Also, findings from Punchihewage-Don et al. [16] (Figure 1) indicate a substantial burden of *Salmonella* in organic and non-organic chickens, characterized by resistance to critical antibiotics and the presence of virulence genes, which together heighten the potential risk of Salmonellosis. From a public health standpoint, contaminated poultry products especially raw or undercooked meat and eggs serve as major sources of *Salmonella* infection in humans. Mishandling, inadequate cooking, and cross-contamination during processing or food preparation also facilitate outbreaks. According to the European Food Safety Authority [17], *S. Enteritidis* remains the leading cause of foodborne outbreaks in Europe, with poultry products frequently implicated. Economically, *Salmonella* infections in poultry lead to direct losses through reduced performance and increased mortality, and indirect losses through trade restrictions, regulatory penalties, recalls, and diminished consumer trust. The increasing emergence of multidrug-resistant *Salmonella* strains from poultry further complicates control efforts and raises significant One Health concerns [18]. Given these risks, reducing *Salmonella* colonization in poultry remains a high priority for both the poultry industry and public health sectors. Moreover, despite the immune system ability to detect pathogens through Pattern Recognition Receptors (PRRs) like Toll-like receptors (TLRs), *Salmonella* may evade detection by the modification of the lipopolysaccharide (LPS) on its surface prevents recognition by TLR4 or downregulate the expression of certain surface antigens that are typically recognized by the immune system [19,20].

The poultry gut and immune system produce various antimicrobial peptides and reactive oxygen species (ROS) which aids in the destruction of invading pathogens. However, *Salmonella* can alter the structure of its outer membrane, reducing the permeability to antimicrobial peptides which are alternatives explored for pathogen suppression [21] while protecting itself from oxidative stress by secreting the superoxide dismutase (SOD) and catalase enzymes [22]. In some cases, *Salmonella* can establish a chronic, asymptomatic infection in poultry by modulating the host immune system and remaining dormant [5], reducing the likelihood of being detected and eliminated by the host immune response. The prevalence of *Salmonella* in host cells and tissues is a significant challenge in poultry populations, however, alternatives to antibiotics, especially probiotics, have emerged as promising tools in this fight, offering a sustainable means to inhibit the multiplication of *Salmonella* species in birds before the chance to colonize or invade the host tissues.

## 3. Probiotics and Their Mechanism of Action Against Salmonella

**Figure 2:**
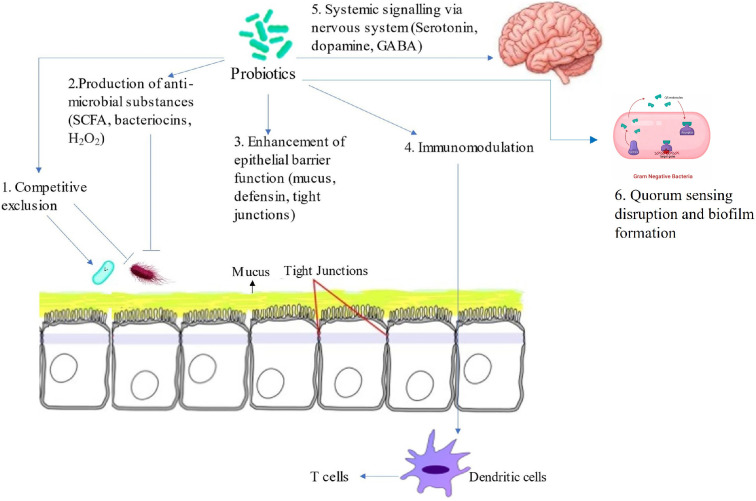
Mechanism of probiotic action against *Salmonella*. Adapted from Latif et al. [30].

Given the limitations of antibiotics and the persistent burden of *Salmonella* in poultry, probiotics have emerged as one of the most promising alternatives for reducing pathogen colonization in birds. Probiotics which are defined as “live microorganisms which, when administered and consumed in food in adequate amounts, confer a health benefit on the host” have gained increasing attention in poultry production due to their potential to reduce *Salmonella* colonization without promoting antimicrobial resistance [8,23]. Importantly, probiotics used in poultry must meet specific criteria including non-pathogenicity, acid- and bile-tolerance, ability to adhere to intestinal epithelial cells, and antimicrobial activity against pathogens [24]. Various bacterial strains, particularly those from the genera *Lactobacillus*, *Bifidobacterium*, *Bacillus*, *Enterococcus* and *Saccharomyces* have been studied for their ability to inhibit *Salmonella* through multiple mechanisms that collectively support host gut health and pathogen exclusion [25]. In poultry production, probiotics are often incorporated into feed or water as supplements to promote the health and productivity of chickens, turkeys, and other birds. Like pathogenic bacteria, probiotics act by colonizing the gastrointestinal tract and influencing various physiological processes that enhance overall poultry performance. Moreover, probiotics exert their benefits through both direct and indirect effects on the host and its microbial environment [26]. Direct effects involve actions that target pathogenic bacteria such as inhibiting *Salmonella* growth, preventing adhesion, or outcompeting pathogens within the intestinal niche while indirect effects, on the other hand, support the host’s natural defenses by enhancing intestinal integrity, modulating immune responses, and improving overall gut resilience. Specifically, as shown in Figure 2, the effectiveness of probiotics in poultry may depend on their ability to inhibit pathogenic bacteria and mechanism of action such as competitive exclusion, enhancement of gut barrier functions, quorum sensing disruption, biofilm formation, modulation of the immune system and the production antimicrobials and metabolites [27,28,29,30].

### 3.1. Competitive Exclusion

One of the primary ways probiotics protect poultry against *Salmonella* is through competitive exclusion. This mechanism involves probiotic bacteria occupying adhesion sites on the intestinal mucosa and competing for nutrients essential for pathogen survival [30]. By establishing early colonization in the gastrointestinal tract, probiotics reduce the ecological niche available for *Salmonella*, thereby minimizing its attachment, proliferation, and translocation across the gut barrier. Early colonization by beneficial bacteria limits *Salmonella*’s ability to establish itself in the gut, a key factor especially in young chicks [31]. Also, when in the gut, probiotics occupy binding sites, consume nutrients, and produce antimicrobial compounds such as lactic acid and bacteriocins which reduce the overall population of AMR-carrying pathogens [25,32] thus limiting the likelihood of gene exchange events. They also acidify the gut by the production of organic acid which lowers the gut pH and creates conditions that are less favorable for plasmid stability and transfer since certain resistance plasmids are less stable or transferable under acidic or nutrient-limited conditions thereby effectively reducing their spread [33].

Several in vivo trials have confirmed reduced *Salmonella* counts following administration of mixed probiotic cultures that effectively outcompete pathogens for adhesion sites and carbon sources [10,34]. Moreover, competitive exclusion is often enhanced by probiotics producing extracellular polysaccharides that strengthen their adhesion to the mucosa [35].

### 3.2. Production of Antimicrobial Compounds

Probiotic bacteria produce a variety of antimicrobial substances that inhibit *Salmonella* growth. Organic acids such as lactic, acetic, and propionic acids acidify the gut environment which consequently reduces *Salmonella* viability [36,37]. Additionally, bacteriocins are synthesized by many probiotics and specifically target *Salmonella* through membrane pore formation or enzymatic degradation [38,39]. Hydrogen peroxide production from certain probiotics also increases oxidative stress which is detrimental to pathogen survival. For instance, a validated hatchery egg disinfection method combined hydrogen peroxide mist (2–3%), ozone, and UV-C to generate antimicrobial hydroxyl radicals, achieving > 5 log CFU/egg reduction of *Salmonella enteritidis* and *typhimurium*. The treatment was effective without impairing hatchability and also reduced other pathogens, demonstrating hydrogen peroxide’s strong antimicrobial potential against *Salmonella* [40]. In vitro and in vivo analyses revealed that *Lactiplantibacillus plantarum* and *Lacticaseibacillus acidophilus* strongly inhibited *Salmonella Heidelberg* (SH), reduced cecal SH loads across all ages, modulated gut microbiota composition, and altered fecal metabolites in ways indicative of reduced oxidative stress, lower intestinal inflammation, and improved gut health, thereby supporting their potential to control SH while reducing antibiotic reliance in broilers [41]. Antimicrobial compounds can lower intestinal pH and directly inhibit *Salmonella* by disrupting its membrane integrity or metabolic functions [42].

### 3.3. Enhancement of Intestinal Barrier Function

The intestinal barrier is a critical defense against enteric pathogens, and probiotics enhance their integrity through multiple mechanisms. Certain probiotics including *Lactobacillus plantarum*, *Lactobacillus rhamnosus* and *Bacillus subtilis* can stimulate goblet cells to increase mucin secretion thereby creating a physical mucus layer that traps pathogens and limits direct contact with epithelial cells [43]. Probiotics also increase the production of tight junction proteins, for instance supplementation with *Bacillus pumilus* or *B. subtilis* significantly upregulated ileal tight junction proteins (occludin, ZO-1, JAM-2), MUC2, and IL-17F on day 14, with sustained expression in the high-dose *B. subtilis* group through day 42 [44]. These proteins form a connection between gut epithelial cells and prevent pathogens such as *Salmonella* from passing through. Also, *Bacillus licheniformis* has been shown to enhance these proteins and improve gut integrity in broilers [45]. Additionally, they also modulate the immune response by increasing anti-inflammatory cytokines such as interleukin-10 (IL-10) while decreasing pro-inflammatory cytokines like IL-6 and IL-8, thus reducing intestinal inflammation and supporting epithelial healing. For instance, different strains of *Lactiplantibacillus plantarum* have been implicated in inflammatory regulation [46,47,48].

Furthermore, probiotics promote a balanced gut microbiota that produces short-chain fatty acids (SCFAs), especially butyrate, which serves as an energy source for intestinal cells and enhances tight junction assembly [9]. Several recent broiler studies have reported improved intestinal morphology, including taller villi and deeper crypts, following probiotic supplementation, indicating enhanced nutrient absorption and gut health [49,50,51]. Together, these effects contribute to a stronger intestinal barrier that reduces pathogen invasion and supports overall poultry performance. Moreover, improved barrier integrity limits pathogen translocation and supports nutrient absorption, which can lead to better growth and health outcomes in poultry.

### 3.4. Modulation of the Host Immune Response

Probiotics are known to also modulate the immune system by enhancing both innate and adaptive responses to *Salmonella* challenge. They act by activating macrophages and dendritic cells which leads to increased phagocytosis and antigen presentation [52]. Additionally, probiotics can enhance the production of immunoglobulin A (IgA) by B cells which neutralizes pathogens, limits their mucosal adherence and promote a balanced immune response capable of resisting infectious pathogens [53,54]. Moreover, anti-inflammatory cytokines like IL-10 and IFN-γ have been increased while pro-inflammatory cytokines like TNF-α and IL-6 reduced by probiotics, resulting in balanced immune activation without excessive tissue damage [55]. Importantly, probiotic supplementation promotes the maturation of gut-associated lymphoid tissue, improving long-term immunity against recurrent *Salmonella* exposure [52]. Some *Lactobacillus* and *Bacillus* strains have even been shown to promote the development of gut-associated lymphoid tissue (GALT) and other immune genes that plays a critical role in immune surveillance [56].

### 3.5. Disruption of Quorum Sensing and Biofilm Formation

Quorum sensing (QS) and biofilm formation are key virulence strategies used by *Salmonella* to persist in the poultry environment. They form protective communities on surfaces like the intestinal mucosa, eggs, and equipment where they resist cleaning and antimicrobial interventions [57]. Studies highlight the role of proteobiotics which are small, secreted metabolites from probiotics like *Lactobacillus acidophilus* and *Bifidobacterium* spp. that interfere with QS systems particularly with the LuxS/autoinducer-2 pathway [58,59]. These compounds act by downregulating the virulence genes associated with the adhesion, invasion, Type III secretion systems, and biofilm formation in *Salmonella typhimurium* and other enteric pathogens [60]. In addition, Lactobacillus species have been implicated in the degradation of *Salmonella*’s autoinducer molecules, suppressing virulence and enhancing pathogen clearance [61].

In poultry, *Lactobacillus reuteri* and *Enterococcus faecium* strains, isolated from poultry guts, have been shown to reduce mucin-mediated adhesion and subsequent biofilm formation of antibiotic-resistant *Salmonella* enterica [62]. This suggests that probiotic-derived QS inhibitors can not only limit surface colonization but also prevent the formation of biofilms that serve as long-term reservoirs for contamination. Moreover, combining such probiotic strategies with physical and chemical approaches has been proposed as a comprehensive barrier against persistent *Salmonella* biofilms in poultry processing environments [57]. While many existing sanitation methods like disinfectants and heat fail to fully eradicate *Salmonella* biofilms, integrating QS-disrupting probiotics could fill critical gaps in current control practices.

## 4. Current Probiotic Strains Used Against Salmonella in Poultry

Probiotics have been studied and applied in poultry production to reduce *Salmonella* colonization and enhance bird health with various bacterial species and strains demonstrating efficacy, either as single strains or in multi-strain formulations, delivered through feed, water, or encapsulated forms to maximize survival and colonization in the avian gut. Below, we review key probiotic genera with demonstrated anti-*Salmonella* activity.

### 4.1. Lactobacillus Species

The genus *Lactobacillus* remains one of the most widely researched probiotic groups in poultry due to its broad range of beneficial effects on gut health and pathogen control. Species including *Lactobacillus plantarum*, *L. acidophilus*, *L. reuteri*, and *L. casei* exert antimicrobial actions primarily through the production of lactic acid, hydrogen peroxide, and bacteriocins that create a hostile environment for *Salmonella* spp. [63,64,65]. These metabolites lower gut pH and competitively exclude pathogens by occupying adhesion sites on the intestinal epithelium [66]. *L. rhamnosus* inhibited the invasion of Caco-2 cells by *S. typhimurium* through the secretion of lactic acid [67]. *Lactobacillus acidophilus* and *Lactobacillus reuteri*, have also been implicated for use in poultry due to their ability to adhere to the intestinal lining, outcompete pathogens for nutrients, and produce antimicrobial substances like lactic acid and bacteriocins which improve antibody production and cell-mediated immunity [68,69]. In mice, *L*. *plantarum* has been implicated in enhancing gastrointestinal and reproductive health, supporting immune modulation, and improving intestinal barrier function [63]. It has also been implicated in improving the gut microbiome and growth performance of yellow feather broilers [65]. In vivo and in vitro, supplementation with *L. plantarum* has consistently demonstrated strong reductions in *Salmonella enteritidis* and *typhimurium* colonization, with some studies reporting reductions of up to 80% in cecal counts [10,70,71]. Notably, *L. plantarum* also improves gut histomorphology by enhancing villus height and crypt depth, which promotes nutrient absorption and growth performance [72].

Other *Lactobacillus* species such as *L. johnsonii* have been reported to reduce *Salmonella* spp. in the mucosa, reduce fecal shedding and increase gut microbiota diversity, thereby enhancing the competitive exclusion of pathogens [73]. *L. casei* supplementation strengthens tight junction proteins in the gut, reducing epithelial permeability and systemic translocation of *Salmonella* [74,75]. Multi-strain probiotic formulations that combine various *Lactobacillus* species have shown synergistic effects, improving pathogen inhibition and host immune responses more than single-strain treatments [76].

### 4.2. Bifidobacterium Species

*Bifidobacterium* species, although less prevalent in poultry probiotics compared to *Lactobacillus*, are gaining recognition for their immunomodulatory and antimicrobial potential. Strains such as *Bifidobacterium breve* and *B. animalis* produce acetic and lactic acids that lower gut pH and secrete bacteriocins effective against *Salmonella* spp. [77]. They also enhance gut barrier function by stimulating mucin production and upregulating tight junction proteins, contributing to pathogen exclusion [78]. *Bifidobacterium bifidum* postbiotics (BbP) protected chickens against *S. Pullorum* infection by regulating pyroptosis, enhancing intestinal barrier function, modulating gut microbiota, and significantly reducing mortality [79]. This highlights BbP’s potential as an effective antibiotic alternative in poultry farming. Additionally, *B. breve* is associated with improved intestinal morphology in poultry such as increase in villus height and goblet cell numbers [80] which leads to improved nutrient uptake and bird health. Another study reported that administering *Bifidobacterium* (B2-2) during late embryogenesis increased bifidobacterial colonization at hatch, reduced Gram-negative bacterial and *Enterococcus* counts, and enhanced early growth performance in broiler compared to non-treated groups [81].

Several in vitro and in vivo studies in other species such as mice, pigs and humans have also revealed antibacterial and antiviral properties of *Bifidobacterium*. For instance, *B. thermophilum* was implicated in the inhibition of rotaviral infection by adherence to Caco-2 and HT-29 cells resulting in low viral titers. Also, pretreatment with *B. thermophilum* led to less epithelial damage of intestinal tissue [82] while porcine intestinal epitheliocytes pretreated with *B. infantis* or *B. breve* showed similar results [83,84]. The growth of vancomycin-resistant *S. aureus* and vancomycin-resistant *Enterococcus* were also inhibited by certain *Bifidobacterium* species including *B. adolescentis*, *B. pseudocatenulatum*, and *B. longum* [84,85].

### 4.3. Bacillus Species

Spore-forming *Bacillus* species such as *Bacillus subtilis*, *B. licheniformis*, and *B. coagulans* are valued in poultry probiotic formulations due to their ability to withstand harsh feed processing conditions and gastric transit [86]. Despite the stability of *Bacillus* spp., inconclusive results were obtained on the performance of disease-challenged broiler [87,88]. However, these species secrete antimicrobial peptides (AMPs), enzymes, and organic acids that inhibit *Salmonella* colonization via competitive exclusion and direct antagonism [89].

Studies have demonstrated that *B. subtilis* supplementation in broilers can reduce intestinal *Salmonella* counts, improve immune parameters (including increased IgA secretion), and promote gut morphology improvements such as increased villus height and reduced crypt depth [90,91,92,93]. Moreover, *B. licheniformis* exhibited strong antimicrobial and anti-biofilm activities against *Salmonella typhimurium* by inhibiting its growth, reducing biofilm formation and components, suppressing motility, and interfering with quorum sensing systems [94]. Another study reported that *Bacillus licheniformis* and *Bacillus subtilis* exhibited anti-inflammatory, antioxidant, and anti-adhesion effects against *E. coli* and *S. typhimurium* in IPEC-J2 cells, suggesting their potential as effective components in probiotic multispecies formulations, despite having minimal impact on paracellular permeability [95]. The spore-forming nature of these probiotics also contributes to their long shelf life and viability under storage, making them commercially viable for large-scale poultry production [96,97].

### 4.4. Enterococcus Species

Certain *Enterococcus* species, including *E. faecium* which has been implicated in improved body weight and reduced mortality rates in poultry [98]. They produce enterocins—bacteriocins with potent activity against *Salmonella* and other pathogens [99]. These bacteriocins can disrupt *Salmonella* membrane integrity, leading to reduced viability [100]. In vivo and in vitro studies show that *E. faecium* supplementation reduces *Salmonella* intestinal colonization, improves gut health, and modulates immune responses by increasing anti-inflammatory cytokines [101,102]. Additionally, *Enterococcus* probiotics contribute to improved feed conversion ratios and weight gain, enhancing production efficiency [9].

## 5. Emerging Probiotic Candidates from Other Hosts Species for Salmonella Control

Exploring probiotic strains derived from non-poultry hosts offers fertile ground for discovering novel interventions against pathogenic bacteria such as *Salmonella*. One promising candidate is *Faecalibacterium prausnitzii* which is primarily isolated from human and ruminant gut ecosystems. Although challenging to culture due to strict anaerobic requirements, field trials in pre-weaned dairy calves demonstrated that oral administration of *F. prausnitzii* significantly reduced severe diarrhea incidence (3.1% vs. 6.8%) and mortality (1.5% vs. 4.4%) while increasing weight gain by approximately 4.4 kg during the pre-weaning period [103]. It also produces anti-inflammatory metabolites including butyrate and salicylic acid which are known to inhibit NF-κB signaling and suppress pro-inflammatory cytokines such as IL-8, IL-6, and IL-12, while promoting IL-10 production and enhancing gut barrier integrity via upregulation of tight junction proteins like occludin and e-cadherin. In poultry gut microbiome studies, *Faecalibacterium* abundance has been associated with improved feed conversion and resilience during pathogen challenge, making it a biomarker and potential functional probiotic in broiler context [104]. In addition, other commensal anaerobes in the Clostridiales order such as *Oscillibacter* spp., and *Megamonas* spp. have repeatedly been associated with reduced *Salmonella* shedding and enhanced gut stability in poultry, with *F. prausnitzii* emerging as a biomarker species in vaccinated layers demonstrating strong resistance to *Salmonella enteritidis* colonization [105].

*Clostridium butyricum* is also a potential probiotic in poultry as it has been widely used in swine and human clinical settings for its butyrate-producing and gut barrier–enhancing properties. In laying hens, dietary supplementation with *C. butyricum* and butyric acid derivatives improved eggshell thickness, lowered pro-inflammatory cytokines (IL-6, IL-8, IL-1β) and modulated ovarian immune function, suggesting systemic benefits beyond the gut [106,107]. It has also demonstrated clear efficacy in broilers since supplementation reduced cecal and systemic *S. enteritidis* burdens, decreased inflammatory cytokines (IL-1β, IL-8, IFN-γ, TNF-α), and downregulated TLR4/MyD88/NF-κB signaling, while improving tight-junction protein expression and microbial alpha diversity in the gut [108]. Although direct trials against *Salmonella* in poultry remain sparse, related studies reveal that combination supplementation of *C. butyricum* in multispecies probiotic formulations effectively reduces *Clostridium perfringens*, *E. coli*, and enhances beneficial microbial populations, pointing toward a broader protective ecological effect [109].

Environmental isolates from fermented foods or poultry caeca microbiota also represents a promising untapped source. A recent animal microbiome meta-analysis identified genera such as *Caloramator*, *DA101* (Ruminococcaceae), *Faecalibacterium*, *Parabacteroides*, and *Solibacillus* as candidate next-generation probiotics negatively associated with *Campylobacter*, and by extension potentially effective against *Salmonella* via competitive exclusion and immune modulation pathways [104]. Notably, isolates of *Lactobacillus casei*, *Ligilactobacillus salivarius*, and *Lactobacillus plantarum* sourced from Tibetan kefir and fermented meats have demonstrated prophylactic efficacy against *S. pullorum* in chicks—reducing mortality to zero, improving growth, elevating cecal *Lactobacillus* counts, and boosting sIgA and IL-4 while suppressing TNF-α and IFN-γ responses [110]. Other core cecal taxa identified across commercial broiler flocks—including *Eisenbergiella*, *Intestinimonas*, *Subdoligranulum*, *Faecalibacterium*, and *Blautia*—are associated with butyrate production, competitive exclusion of pathogens, and overall gut resilience, marking them as candidate next-generation probiotics. Moreover, complementary strains such as *Enterococcus faecium*, isolated even from poultry feces, have shown efficacy in reducing *Salmonella* colonization and enhancing immune markers when used in multi-strain formulations [111].

Furthermore, recent studies have begun to spotlight microbial strains originally isolated from non-poultry hosts or environmental niches that exhibit compelling anti-*Salmonella* and gut health-promoting properties in poultry. For instance, Mix10 consortium derived from feral chicken ceca, which includes *Olsenella* sp., *Megamonas funiformis*, *Pseudoflavonifractor* sp., *Faecalicoccus pleomorphus*, and others significantly reduced *S. Typhimurium* colonization and curtailed expression of pro-inflammatory mediators including NFKB1, MAPKs, and IRFs, while supporting anti-inflammatory immune modulation in gnotobiotic and conventional chick models [112].

Collectively, these findings support a promising direction for probiotic discovery as leveraging strains from diverse hosts and environments such as human-derived *F. prausnitzii*, swine-adapted *C. butyricum*, feral chicken cecal consortia, and isolates from fermented foods—may yield probiotic candidates with robust anti-*Salmonella* activity, improved gut barrier function, and immunomodulatory capacity. Rigorous validation in poultry-specific challenge experiments including colonization stability, metabolic profiling like SCFA production, immune outcomes, and ecological impact will be essential to translate these candidates into practical, safe, and effective probiotic tools for poultry health management.

## 6. Challenges in Probiotic Use in Poultry

Long-term probiotic use in poultry production presents complex dynamics, particularly regarding horizontal gene transfer (HGT) and antimicrobial resistance (AMR) mitigation. Probiotics contribute to reducing pathogen load via competitive exclusion mechanisms by occupying mucosal binding sites, competing for nutrients, and producing antimicrobial metabolites such as lactic acid and bacteriocins suppressing the population of AMR-carrying pathogens [32], consequently reducing the frequency of gene exchange events within the gut ecosystem. Moreover, the acidification of the gastrointestinal tract through organic acid production creates an inhospitable environment for plasmid stability and conjugative transfer, as certain resistance plasmids exhibit reduced stability under acidic or nutrient-limited conditions [33]. Additionally, some probiotic strains interfere with quorum sensing—disrupting bacterial communication systems that regulate gene expression and plasmid conjugation, thereby limiting conjugative plasmid transfer events [59]. Enhanced gut barrier integrity and mucosal immunity, particularly the stimulation of secretory IgA, further inhibit interactions that facilitate gene transfer among pathogenic bacteria [113].

However, recent studies emphasize that long-term administration of probiotics can induce unintended ecological shifts within the gut microbiota. Persistent use of a single probiotic strain may lead to its dominance, suppressing beneficial commensals and reducing overall microbial diversity, which could increase susceptibility to opportunistic infections [114]. Furthermore, probiotics that are not thoroughly characterized may carry antimicrobial resistance genes located on mobile genetic elements, posing risks of horizontal gene transfer to resident or transient pathogens [115]. A comprehensive metagenomic analysis of commercial poultry probiotics revealed the presence of diverse ARGs including fluoroquinolone, macrolide, and aminoglycoside resistance determinants such as *aadK*, *AAC(6′)-Ii* and multiple drug-resistance genes (*vmlR*, *ykkC*, *ykkD*, *msrC*, *clbA*, *eatAv*), highlighting concerns about inadvertent resistome enrichment, even in the absence of observable resistance expansion over a 60-day period [116]. Additionally, the efficacy of probiotics under pathogen challenge conditions appears highly context specific. For instance, trials with *Bacillus subtilis* QST713 in broilers infected with *Clostridium perfringens* demonstrated improvements in growth performance and gut barrier integrity [117], but the outcomes may significantly be influenced by the challenge model, strain specificity, and administration route. Similarly, in vitro and in-vivo evaluations of dual-strain probiotics across multiple broiler flocks revealed variable efficacy in mitigating mild necrotic enteritis, underscoring the necessity of strain-specific optimization [118,119]. Challenges also persist in probiotic viability during processing and delivery; a recent study comparing freeze-dried and spray-dried formulations of lactic acid bacteria including *Enterococcus faecium*, *Ligilactobacillus salivarius*, *Pediococcus acidilactici*) demonstrated high survivability (>94%) and beneficial effects on gut morphology, yet strain-dependent variability in gastric resilience and colonization capability was evident [111]. Collectively, these findings indicate that probiotic efficacy in poultry is strongly influenced by strain selection, dosage, route and timing of administration, and environmental or management conditions. Strain-specific attributes govern antimicrobial activity, immune modulation, and intestinal colonization capacity, whereas dosage and formulation determine microbial viability and persistence during processing and delivery. Moreover, variability in challenge models, baseline gut microbiota composition, production systems, and environmental stressors substantially contributes to the inconsistent outcomes reported across probiotic intervention studies.

Beyond individual strain concerns, broader ecological implications must be considered. While probiotics are intended to stabilize gut health and reduce pathogen shedding, their long-term impacts on the gut microbial community and ARG dynamics are not fully understood. Systematic reviews indicate that probiotic supplementation can inconsistently affect AMR gene reservoirs, with outcomes influenced by baseline microbial diversity, host factors, and environmental variables [120,121,122]. Furthermore, the baseline AMR burden in different production systems (conventional, antibiotic-free, organic) varies greatly, complicating the uniform application of probiotic strategies across diverse farming contexts [123,124].

Consequently, these findings underscore that although probiotics remain a promising alternative to antibiotic growth promoters, issues related to strain safety, ARG carriage, formulation viability, challenge-specific efficacy, and ecological consequences require continuous monitoring and rigorous evaluation within real-world production environments.

## 7. Future Directions in Probiotic Application for Poultry

To overcome current challenges and optimize the sustainable use of probiotics in poultry production, adopting a multidisciplinary approach that integrates advanced molecular tools, targeted application strategies, and comprehensive field validation is expedient. One of the foremost priorities is the integration of microbiome profiling to guide precision probiotic interventions. High-throughput sequencing technologies such as 16S rRNA gene amplicon sequencing and shotgun metagenomics, can provide detailed insights into the baseline gut microbiota composition and functional potential of different poultry populations [125]. Such data will enable the development of tailored probiotic formulations that complement the existing microbiota, thereby enhancing colonization efficiency and functional efficacy.

Beyond compositional profiling, the use of genomics and metabolomics for strain selection and functional characterization is essential to ensure safety and efficacy. Whole-genome sequencing of candidate strains can identify beneficial traits, such as bacteriocin production, adhesion factors, and immunomodulatory properties, while simultaneously screening for the absence of virulence factors and mobile antimicrobial resistance genes [106,125]. Metabolomic analyses can further unravel the metabolic outputs of probiotic strains, such as short-chain fatty acids and organic acids. In addition to conventional probiotics, emerging biotic strategies such as synbiotics (probiotic-prebiotic combinations), paraprobiotics (non-viable inactivated probiotics), and postbiotics (metabolites and cell components derived from probiotics) are potential complements or alternative approaches [126]. These next-generation formulations may help to overcome some of the limitations associated with strain viability and colonization while retaining functional benefits such as pathogen inhibition, immune modulation, and gut barrier enhancement. To ensure reproducibility and translational applicability, there is a pressing need to standardize efficacy testing protocols and develop robust regulatory frameworks that govern probiotic use in poultry [127]. Standardization should encompass not only strain identification and safety evaluation but also functional performance metrics under controlled and field conditions.

Furthermore, the translation of laboratory findings to real-world applications necessitates long-term, large-scale field trials across diverse production systems and geographic regions. Such trials are critical for capturing the complex interactions between probiotics, host genetics, management practices, environmental factors, and the farm-specific microbial ecosystem [128]. Data generated from these long-term studies will be invaluable for refining probiotic formulations, informing best practices, and embedding probiotic use within comprehensive antimicrobial resistance mitigation frameworks aligned with the One Health approach.

## 8. Conclusions

Probiotic interventions offer a promising alternative to antibiotics for mitigating *Salmonella* infections in poultry with growing scientific evidence supporting their role in improving gut health, enhancing immunity, and reducing pathogen colonization. Moreover, while strains like *Lactobacillus*, *Bacillus* and *Bifidobacterium* have shown consistent benefits, emerging candidates from other animal hosts could offer broader or more targeted effects particularly in the presence of zoonotic infections like *Salmonella*. However, the efficacy of probiotics depends heavily on strain selection, dosage, administration route, and environmental conditions. In addition, significant challenges regarding probiotic application in poultry remain, including variability in outcomes across studies, lack of standardized regulations, and limited understanding of host-microbe interactions in diverse poultry production systems. Future research should focus on the development of next-generation probiotics, better characterization of strain-specific actions, and integration with other health-promoting strategies like phytobiotics and prebiotics.

It is important to optimize probiotic use as it holds great potential not only for improving poultry health and productivity but also for enhancing food safety and addressing global concerns related to antibiotic resistance.

## Data Availability

No new data were created or analyzed in this study. Data sharing is not applicable to this article.
